# *Wake up, wake up! It’s me! It’s my life!* patient narratives on person-centeredness in the integrated care context: a qualitative study

**DOI:** 10.1186/s12913-014-0619-9

**Published:** 2014-11-29

**Authors:** Geva Greenfield, Agnieszka M Ignatowicz, Athina Belsi, Yannis Pappas, Josip Car, Azeem Majeed, Matthew Harris

**Affiliations:** Department of Primary Care and Public Health, School of Public Health, Imperial College London, The Reynolds Building, St Dunstan’s Road, London, W6 8RP UK; Division of Health Sciences, Warwick Medical School, The University of Warwick, Coventry, CV4 7AL UK; Department of Surgery and Cancer, 2nd Floor, Paterson Centre, St Marys Hospital, Imperial College London, South Wharf Road, London, W2 1BL UK

**Keywords:** Integrated care, Patient centered-care, Patient experience, Patient empowerment, Shared decision-making, Partnership, Interview, Qualitative study

## Abstract

**Background:**

Person-centered care emphasizes a holistic, humanistic approach that puts patients first, at the center of medical care. Person-centeredness is also considered a core element of integrated care. Yet typologies of integrated care mainly describe how patients fit within integrated services, rather than how services fit into the patient’s world. Patient-centeredness has been commonly defined through physician’s behaviors aimed at delivering patient-centered care. Yet, it is unclear how ‘person-centeredness’ is realized in integrated care through the patient voice. We aimed to explore patient narratives of person-centeredness in the integrated care context.

**Methods:**

We conducted a phenomenological, qualitative study, including semi-structured interviews with 22 patients registered in the Northwest London Integrated Care Pilot. We incorporated Grounded Theory approach principles, including substantive open and selective coding, development of concepts and categories, and constant comparison.

**Results:**

We identified six themes representing core ‘ingredients’ of person-centeredness in the integrated care context: “*Holism*”, “*Naming*”, “*Heed*”, “*Compassion*”, “*Continuity of care*”, and “*Agency and Empowerment*“, all depicting patient expectations and assumptions on doctor and patient roles in integrated care. We bring examples showing that when these needs are met, patient experience of care is at its best. Yet many patients felt ‘unseen’ by their providers and the healthcare system. We describe how these six themes can portray a continuum between having own physical and emotional ‘*Space*’ to be ‘seen’ and heard vs. feeling ‘*translucent*’, ‘unseen’, and unheard. These two conflicting experiences raise questions about current typologies of the patient-physician relationship as a ‘dyad’, the meanings patients attributed to ‘care’, and the theoretical correspondence between ‘person-centeredness’ and ‘integrated care’.

**Conclusions:**

Person-centeredness is a crucial issue for patients in integrated care, yet it was variably achieved in the current pilot. Patients in the context of integrated care, as in other contexts, strive to have their own unique physical and emotional ‘space’ to be ‘seen’ and heard. Integrated care models can benefit from incorporating person-centeredness as a core element.

*“The good physician treats the disease; the great physician treats the patient who has the disease”*

Sir William Osler (1849–1919)

## Background

Patient-centered care is a multifaceted construct that can be defined according to different sociological theories, including functionalism, conflict theory, utilitarian theory, and social constructionism [1]. We prefer using here the term ‘person-centeredness’ instead of ‘patient-centered care’, to emphasize the ‘personhood’ of people in care rather than their sick role. The literature includes several definitions of what constitutes person-centeredness [2-8]. A recent review on patient-centered care in chronic disease management identified six major themes including ‘starting from the patient’s situation’, ‘legitimizing the illness experience’, ‘acknowledging the patient’s expertise’, ‘offering realistic hope’, ‘developing an ongoing partnership’, and ‘providing advocacy for the patient in the healthcare system’ [9]. Understanding the term simplistically and literally, person-centeredness is all about putting patients first, at the center of health and social care, that is respectful and responsive to individual patient preferences, needs and values. Patient-centeredness is now considered a core element of high-quality healthcare [10] and is generally related to higher quality of life, lower anxiety and depression [11]. There is evidence to show that outcomes in diabetes improve when patients take an active role in their care. These outcomes include improved wellbeing, better communication with the doctors and greater treatment satisfaction [12], better blood sugar control and less functional limitations [13]. Obstacles to treatment adherence, are common across countries, and seem to be related less to issues of the health-care system and more to patient's knowledge about diabetes, beliefs and attitudes and the relationship with health-care professionals [14]. A systematic review on the effects of modification of provider-patient interaction and provider consulting style on patient diabetes self-care and diabetes outcomes summarized that enhancing patient participation in care show good efficacy and efficiency, and improve patient self-care and diabetes outcomes, and are more effective than changing provider behaviour into their into a more patient-centred consulting style [15]. Generally, trials demonstrated that improved physiological measures (blood pressure or blood sugar), behavioural measures (functional status), and subjective measures (evaluations of overall health status) were consistently related to specific aspects of physician-patient communication [16,17].

Person-centeredness can be viewed as a part of a visible trend in the last three decades of growing patient expectation to be treated as a whole person and to be engaged in decisions about their healthcare. The concepts of empowerment, enablement, person-centeredness, shared decision-making, partnership, choice and preferences, all emphasize the centrality of the patient in medical care. They highlight the attention to patients’ psychosocial as well as physical needs, listening to the patient ‘story’ beyond their clinical concern, and empowering patients to take ownership on their own health. Shifts from the traditional biomedical, paternalistic model to more symmetrical models emphasize the joint negotiation and partnership between physicians and patients, empowering patients to take a proactive role in their care [18,19]. This has emerged alongside other concurrent evolvements: patient roles have become more diverse, and the doctor-patient relationship has become multifaceted and more pivotal to health outcomes [20]. Medical consumerism situates the patient as a consumer of health services [21,22], with the ‘privileges’ of choice and say. Clinical information accessible online have partially flattened the knowledge dominance of the physician, allowing patients greater engagement with managing their health and in the patient-physician relationship [23]. The traditional paternalistic model assuming the physician solely have the decision making ability, has been replaced by more symmetrical models, that emphasized the joint negotiation and partnership between physician and patient [24-27].

### Person-centeredness and integrated care

Another timely approach emphasizing putting patients first, though stemming from a different perspective to patient-centeredness is integrated care. Integrated care emphasizes alleviating the patient journey through health services and empowering health professionals through improved coordination of care, aiming at improving health outcomes and reducing healthcare costs [28].

One way to theoretically look at integrated care is from a structural functionalist view, seeing society (a local health economy in our case) as a complex system whose parts work together to promote solidarity and stability (i.e., by facilitating coordinated, join-up work of clinicians working in different settings and levels of care). This is a macro-level view on structures and organizational mechanisms that support coordinated care. On the other hand, ‘person-centeredness’ can be perhaps viewed from an interpretivist or interactionist view, emphasizing subjective meanings of micro-scale social interactions, such the doctor-patient relationship. Hence, although person-centeredness and integrated care have seemingly a similar ethos, they come from different agendas and theoretical frameworks.

Some attention has been given to the importance of person-centeredness in integrated care [29,30]. Several bodies have been contributing to the definition of person-centredness in the integrated care context, such as the International College of Person-centred Medicine, the Kings’ Fund in the UK, the Picker Institute, the International Foundation for Integrated Care, and the International Alliance of Patients’ Organizations, the Patient-Centered Outcomes Research Institute, and others. For example, The *International Journal of Integrated Care* dedicated in 2010 a whole volume on person-centered medicine [31]. There is also an evidence that a person-centric integrated care pathway improved rehabilitation outcomes and cost-effectiveness [32]. Yet, person-centeredness is being implemented in mainstream provision in integrated care models in only 2 out of 9 European countries [30]. Several systematic reviews have shown that user views are rarely evaluated in integrated care programs [33,34], and that patients are rarely involved in designing, implementing or evaluating patient-centered care interventions [35]. Patients with long-term conditions often struggle to navigate through corridors of hospitals, as a metaphor of navigating through mazes of fragmented healthcare systems.

While there is an acknowledgement of the importance of patient engagement and person-centeredness, taxonomies of integrated care focus on functional, organizational, professional, and clinical integration [36], but rarely on person-centeredness [30,33]. Taxonomies of integrated care, such as ‘vertical’ and ‘horizontal’ integration [36] neither define around what, nor whom, care is being integrated. They certainly do not imply that the care is being integrated around the patient. Hence they mainly describe how patients fit within services that are integrated, rather than describing how services fit into the patient world. Such an approach might neglect the patient agenda, situating the patient as a passive, almost translucent recipient of integrated services.

In the UK, the Department of Health published several reports setting out the Government’s long-term vision to put patients “at the heart of everything we do” [37] and the “No decision about me, without me” idea [38]. Yet, shared decision-making is currently far from being a norm in the NHS [39]. In a recent survey of 16 UK integrated care pilots, most patients felt uninvolved in decision making regarding their care [40].

### Goals

Patient-centeredness has been commonly defined through physician’s behaviors aimed at delivering patient-centered care [1], yet patient narratives of person-centeredness, particularly in the integrated care context, are scarce, and it is unclear what ‘person-centeredness’ means in integrated care through the patient voice. Very few attempts have been made to explore patient views on integrated care [41,42]. The literature highlights a room for improvement in person-centeredness in integrated care models. It is first useful to understand what ‘person-centeredness’ means for patients in the integrated care context, and whether integrated care is delivered to them in a person-centered manner. We hence aimed here to explore patient narratives on person-centeredness in the context of integrated care.

### Why this is important? Why now?

Exploring person-centeredness in the integrated care context is important from two perspectives: the micro perspective (patient) and the macro perspective (public health). First, patients nowadays increasing expect to receive more person-centered care. While this is not applicable to all patients, many patients would like to be more involved in their care [43], situating themselves as proactive partners to their providers. Additionally, the current era of medical consumerism and access to medical knowledge on the internet, emphasizes patient demand to receive the best available care and their greater knowledge on their conditions and available treatments. Second, from the public health perspective, long-term conditions are currently the leading cause of mortality worldwide [44]. Patient-centeredness is widely acknowledged as a core element of high-quality healthcare tacking long-term conditions [10,45]. Patients with long-term conditions have multiple medical, physical, psychological, and social needs, requiring a mix of services provided simultaneously by multiple providers, in the home, community and institutional settings. Their care requires different models of care that coordinate and integrate professions and institutions from different settings and levels of care. Hence there is currently wide international acknowledgment for the need to initiating new models of care designed to alleviate coordination between professionals and services.

## Method

This study was a part of a large mixed methods evaluation of the Northwest London Integrated Care Pilot, probing its clinical, organizational and financial impact. A detailed description of the intervention and its evaluation appears elsewhere [46,47]. The program was launched as a pilot intervention in June 2011. The service aims at providing integrated care for the elderly and those with diabetes in a population of 550,000 people. The program link more than 100 general practices, two acute care trusts, five primary care trusts (now called Clinical Commissioning Groups), two mental health trusts, three community health trusts, five local authorities, and two charities. Core interventions are (1) proactive care planning incorporating both health and social care, (2) case discussions at multi-disciplinary professional groups, including GPs and acute, mental health and social care specialists, and (3) an information-sharing technology tool.

### Design

We conducted a phenomenological, qualitative study to explore patient narratives on person-centeredness in the integrated care pilot. Phenomenological research aims to describe an experience, and hence a suitable method for the study question [48,49]. We conducted semi-structured, open-ended personal interviews with patients. Such interviews are systematic yet sensitive to the dynamics of the conversation. We did not intentionally target person-centeredness; the interest in it rose from the data, when patients predominantly raised issues around person-centeredness.

### Interview protocol

The interview questions emerged from the relevant literature and previous non-participant observations and focus groups conducted by our team. The interview focused on patient narratives and experience in their current care, including access, satisfaction from care and providers, continuity of care, empowerment and shared-decision making, care provision, organization, and integration. We intentionally did not use the term ‘person-centered care’ as it is an academic term, and we wanted the participants to decant their own meanings. We asked them what aspects of care are the most important to them, what can be improved, and what the best and worst aspects of their care are. Along the protocol, we prompted with spontaneous questions, using clinical interview techniques, such as reflection, restatement, clarification, and exploration. The interviews were audio-taped and transcribed verbatim while ensuring anonymity. The interview was designed to fit a period of 60 minutes. The protocol was similar across all interviews, with some adjustments to the order of questions.

### Participants and sampling

The participants were people with diabetes and/or people over 75 years old who were registered with the pilot. We interviewed a purposive sample of 22 patients, who were contacted through several GP practices in North-west London, and through the pilot’s Patient and User Committee. Twelve of them (55%) were male and 10 were female and all of them were above 50 years old. The interviews took place between May and July 2012 at the patients’ clinics, or at the Department of Primary Care and Public Health at the Charing Cross Hospital Campus of Imperial College London. The protocol was approved National Research Ethics Service Committee for City and East London (Ref. 11/LO/1918). We removed any identifying details from quotes brought here, such as providers’, clinics’ and hospitals’ names. Patients were compensated for travel expenses in accordance to the NIHR INVOLVE guidelines.

### Data analysis and thematisation

While we did not perform a Grounded Theory approach *per se*, we adopted its basic principles, including minimizing preconceptions without predetermined research “problem”, substantive open and selective coding, and constant comparison [49]. In the Grounded Theory approach, codes, concepts and categories stem from the data itself without preconceived expectations. Thorough coding process conducted by qualitative researchers (GG, AI) who independently analyzed the interviews and developed the basic codes. We developed the codes both ‘horizontally’ (by coding each interview as a standalone hermeneutic unit) and ‘vertically’ (by scanning across the interviews for specific terms, e.g., ‘patient’, ‘person’, ‘care’, ‘preference’, ‘empowerment’, ‘engagement’, ‘decision’, ‘attention’, etc.). We then identified themes (known as ‘concepts’ in the Grounded Theory approach) based on the open codes. We then developed these themes into broader themes (known as ‘categories’ in the Grounded Theory approach). We worked in a spiral process in which concepts led to creation of new codes and vice versa. We used mind mapping techniques to visually arrange and conceptualize the codes, concepts and categories, their structure and linkages. We conducted on-going discussions on coding, themes and interpretations. While analyzing the data we examined our own roles and possible biases (reflexivity). As some of the research team were social scientists (GG, AI, AB, YP), we come from our perspective on integrated care, person-centeredness and the patient-physician relationship from our experiences as patients. Working together with clinicians (MH, JC, AM) assured a balanced view accounting for clinical perspectives. Data collection and coding continued until we reached theoretical saturation, i.e., when new information produced little or no change to coding and thematization. Full thematic saturation was reached towards the last interviews, reflecting the complexity of the narratives. We coded and analyzed the data using the Atlas.ti® software version 7.

We aimed to refine core ‘ingredients’ of person-centeredness as reflected from the participants, i.e., what constitutes ‘person-centredness’ in the integrated care context. We wished to create a model which is parsimonious yet including different dimensions of person-centredness. We compared elements emerged from the thematic analysis to the theoretical underpinning of concepts previously described person-centred care, yet not specifically in the integrated care context (e.g. shared-decision making, continuity of care etc.).

## Results

**Figure 1:**
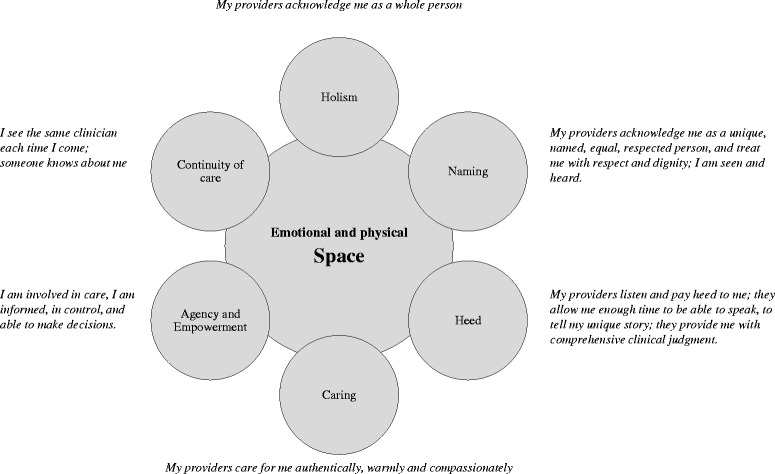
**Patient narratives on person-centeredness in the integrated care context.**

Each patient brought their personal world and unique narrative to the interviews. By allowing them to freely talk about their care, we tried to reveal the fine ingredients that make patients feel in the center of their care. The narratives included their worries, hopes and fears, some major life events, their diseases, their social situation, their daily coping with long-term conditions, and some early views into end-of-life. The narratives reflect their experience as being a patient in the NHS, and their beliefs about what care should be. The narratives conveyed satisfaction from a close, warm encounter with a clinician, but also deep frustration with the system. We identified six themes or ‘ingredients’ of person-centeredness, including “Holism”, “Naming”, “Heed”, “Compassion”, “Continuity of care”, and “Agency and Empowerment” (Figure 1). There are overlaps between the different themes as all of them refer to patients’ expectation to be the center of their care. We then identified an overarching theme interlacing the six ‘ingredients’ of person-centeredness, thematized as the experience of having their own physical and emotional ‘Space’ vs. not having such as space. We will describe these themes below.

### Holism: I want to be treated as a whole person

The participants expressed a prominent expectation to be ‘seen’ as a whole person with a whole life beside their medical symptoms, and having psychological as well as medical needs. In the first quote, viewing the physician as a whole person, not just a medical expert, helped the patient to build a trusted relationship. The other, emotionally loaded quote exemplifies how the patient felt “*wiped out as a whole person*” when a physician noted, apparently insensitively, that the patient did not meet their clinical goals, reflecting a view of the patient as a clinical success or failure, and the need “*to separate the condition from them*”:

*“I mean, I think they know… they know I was…****not just about my diseases****, but they know about my family life and I know about the doctor’s children and, you know. I mean, it’s more like, you know, I… really, I count them as friends, to tell you the truth…****It’s always a pleasure to come here”****. (Participant 21)*

*“…****It wipes me out as a whole person****……****there’s so much judgment in that, I feel punished****…****because they’re giving me no respect at all****and they’re just saying your HPA is too high…What they need to do is they need to talk about my condition but treat me as a… [person – GG]…****they need to separate the condition from me****”. (Participant 17)*

A holistic approach is otherwise attentive to psychological needs and viewing the patient as a whole body-soul entity. Patients with long-term conditions commonly have unique psychological needs, as shown by the first quote. We exemplified below a positive situation, where the physician attended to psychological needs of the patient, giving them a unique space on top of medical symptoms, made a completely different experience for the patient:

*“…Talking about mental health I also avail of psychotherapy, psychotherapeutic support, but I pay for that privately…****It’s very important for diabetics to be able to talk. That’s one of the big, big problems with diabetes is that diabetics they don’t, they feel isolated; they don’t think anybody understands”.****(Participant 17)*

*“I was sitting there and it was for totally something different, and he said to me, what are your moods like? and I just looked at him and I went you just don’t want to know. And he went,****make me double appointment for Monday, he said and I want to see you Monday****, and that’s when he picked up on it. But since then it’s not better, but it’s easier. I think it’s because maybe that****somebody actually sort of saw how I felt and recognized how I felt****and I wasn’t feeling like I was going mad, … and that’s when he said that I was suffering with depression”. (Participant 2)*

### Naming: I want to be acknowledged as a unique, respected, equal person

A core need, and a source of major frustration expressed by patients, is their wish to be acknowledged as a unique person, ‘not like a number’, and as such, to be treated with respect and dignity. The following quotes depict these needs, and carry heavy emotional tone, reflecting the frustration patients experience when they feel ignored, disrespected, or taken for granted. A common complaint was at bad manners of both physicians and administrators, using professional dominance and patient dependency to excuse themselves from basic human manners. Other than mere bad manners, patient experienced arrogant and patronizing attitude of some clinicians and administrators:

*“Well, feeling that you’re just one of… There was one doctor that I saw who looked at the screen and just, with no conversation, was just yawn, yawn, non-stop yawning and I said excuse me, am I boring you? I hadn’t said anything.****So, I thought no, I’m on to a loser here. I don’t know why I’m here!****” (Participant 3)*

*(The doctor just asks:) “‘Tell me, what can I do for you?’ Sometimes, and I feel bad about that, I am waiting for the appointment, for the doctor to see me, and he comes out several times,****he doesn't even say hello****, right? I think he knows me for a while, and it is a matter of courtesy to say hello”. (Participant 7)*

*“My biggest complaint is that bureaucracy business. If only,****if only they would take time to think that they’re talking to people****and people want to know what’s happening to them, instead of this terrible silence between even booking clerks who make appointments for you.****I mean they’re talk to you as if you’re a number****!” (Participant 5)*

The next quote deserves a special attention. The patient talked about the feeling of being left for months without knowing what is happening with their treatment:

*“I think the bureaucracy of the National Health booking system needs an overhaul…if they were able to give you more information at the time****instead of leaving you for months not knowing what’s happening****… I mean, when I’ve been getting treatment it’s been first class. It’s just the lack of…****It’s infuriating, because you get so fed up with them you want to scream at them sometimes: wake up, wake up! It’s me. It’s my life****”. (Participant 5)*

We chose the quote *“Wake up, wake up! It’s me! It’s my life!”* for the title of the manuscript as it encompassed an overall account in the interviewee’s narratives. It is like the patient was calling the system to *wake up* and revisit its current attitude, and the need to realize a change in patient expectations from care. In the words *“It’s me! It’s my life!”* the patient called the system to realize that the patient is the one that should be the center of care. It is a call for a ‘Space’, for acknowledgment, for realizing that people’s lives are at stake.

### Heed: I want to be listened to and get proper attention

Patients expressed a prominent need to be listened to and to get full attention, both clinically (getting comprehensive clinical judgment), and personally (having enough time to speak and tell their unique story). This attentive listening and attention was important for many patients predominantly because they want to be understood. A common complaint expressed towards providing superficial clinical judgment, tackling merely immediate clinical symptoms, and offering ‘quick fix’ solutions such as painkillers, while unheeding a broader perspective on the patient medical problems. The interviewees were mostly satisfied with waiting for a couple of days for an appointment; yet they were frustrated that after waiting a couple of days, they felt that the physician did not pay full heed to them:

*“Well, I don’t know what to say, but I think it’s a waste of time, because whenever you come to see the doctor, you come for any pain, right, or any problem.****Always it’s have some paracetamol****,… you come with the hope that you’re going to be seen by the doctor or sent to another specialist or something like that, and then the doctor tells you that. It’s disappointing”. (Participant 7)*

Along a proper clinical judgment, patients expected an unrushed, attentive space during the visit. Although not all, many patients felt that that physicians rush to move quickly to the next patient, and felt being dismissed without a proper heed. This space is especially important for patients with multiple conditions, who require attention for each condition separately and as a whole. They need time and attention to be able to speak and tell their unique story. The following quotes bring positive and negative experience of having this unrushed space:

*“It’s partly because of the rapport and partly because he listens.****He has time.****He doesn’t… I don’t see the note and I don’t see the prescription pad on the desk with the pen at the ready”. (Participant 4)*

*“Well, the idea is, because, is, every time we go to GP, you are rushing, so queue behind, you have 20, 30 people.****Everything is angry in the waiting rooms…you are under pressure, always that…You don’t have enough time with your GP to talk****, to discuss your problems…”. (Participant 20)*

### Compassion: I want to be cared for with authentic empathy and warmth

This element refers to soft qualities of empathy and warmth, and feeling that providers authentically *care* of them. Some patients expected their relationship with their physicians to extend from mere scientific medical care to a more personal, less formal relationship. The first two quotes below reflect disappointment from feeling uncared. In the third quote, the patient tried to describe the meaning of care by parallelizing medical care to the parent-child relationship:

*“****Care, the word care, says it all****. Care is care, and if you are in the care of somebody, you do things for them. You try to improve, right? It’s like if I know that person is in my care, the first thing I will feel for that person is****love and compassion****because he is an elder, because he cannot do the things herself, or himself, fragile and all these things, and the same happens with us… I think the doctors,****they don’t care, right?****They are so busy, they have their own lives, of course, but if you have that profession, it’s because you love it, and then****you have to love your patients****, right?” (Participant 7)*

*“They all think they’re doing a good job for themselves,****but they don’t care about who they’re doing it for****”. (Participant 5)*

### Agency and empowerment: I want to be involved in my care

A notable pattern along the interviews was how most of the patients absorbed concepts of paternalism and presumptions of the unequal nature of the doctor-patient relationship, e.g.:

*“I just tell him what I feel, and the****doctor will tell me whatever is good for my health…****” (Participant 9)*

*“She listens to what I have to say and then she answers the questions and****tells me what to do****”. (Participant 15)*

*“****Well, it’s not for me to say it’s… they know better than me****”. (Participant 11)*

*“My GP is kind of trying to****protect me****from what is happening” (Participant 20)*

This absorption was evident not only from what they said (i.e., the content) but from how they said it (i.e., the form), using passive language, almost describing themselves as passive objects operated by health providers, e.g.:

*“When he discharged me…” (Participant 4)*

*“I’d been referred…Now I’m waiting to find out… what they intend doing now”. (Participant 5)*

*“…They wanted me to come … at two o’clock”. (Participant 15)*

Most of them, when talking about ‘involvement’, actually referred to ‘compliance’, i.e., being involved in the treatment mean to comply with the doctor’s orders. This might have been a result of the sample, consisting of older patients educated on the paternalistic model. The phrase ‘*They know better than me*’ (Participant 11) proves this generational difference. Few patients did express more proactive perceptions of their role as patients. Few patients referred to their agency and their need to take proactive role in the term, instead of expecting their GP to do so for them:

*“****Would I like to be more involved****; I would like to be able to ask my doctor that I’ve heard about such and such medication or project or research program, or whatever, and I would like to be able to ask my doctor that is it possible for me to avail of this and find out more about it and what’s going on and, maybe, even partake in it”. (Participant 17)*

*“****I want to be close to the doctor****. At least they explain to me everything that I can understand, and I can ask a question for that”. (Participant 16)*

### Continuity of care: I want to be seen by the same doctor each time

Most of the participants preferred to see the same physician each time they visit their clinic. This continuity is important for establishing a trusted relationship with a regular clinician, but also for being treated by a clinician who knows their medical history and hence can see coherent clinical picture. The following quotes show how important this continuity can be for patients*:*

*“…Okay, you’re not going to believe this but one of the most maddening thing of all is you go into the GP’s surgery and there’s about ten different GPs in the practice,****you don’t know who you’re going to see, you know, different doctors coming and going****, and I felt …****if something goes wrong now, who really knows about me****?”*

The patient continued:

*“****I felt I might as well just not have gone to the doctor****…****if a doctor doesn’t know you at all****, you go in. Now, you don’t want to talk to the doctor because the doctor’s staring ahead, looking at the screen and you don’t know whether to interrupt them, to say what is wrong with you or to let him try and read, quickly, what is wrong, which isn’t going to get all that information, that quick, when he hasn’t seen you before, …****It makes such a difference if you’ve got a doctor that you know****…” (Participant 3)*

### Putting it all together: person-centeredness at its best

When these needs described above are met, patient experience of care is at its best. What we as researchers call ‘person-centeredness”, patients simply attribute to holistic, respectful, attentive, compassionate, continuous, and empowering encounters with their health providers:

*“…Because when you come it’s pleasant. They speak to you and talk to you and they ask you what your problems are and then the doctor is the same. She takes a little time and that she’s very sweet, goes through all your things very carefully, and that’s what I want”. (Participant 15)*

*“…It’s good, yes, it is…the receptionist, like she does the blood and everything as well, the nurse, you know, she, you come in and she’s not like the nurse on reception,****she’s like your friend,****you know, you can chat to her, and we’ve chatted about anything and everything, but****she don’t feel like a nurse****”. (Participant 2)*

### An overarching theme: ‘Space’

After identifying these six ‘ingredients’ of person-centeredness, we thought it would be helpful to create an overarching theme, describing how these ‘ingredients’ can be theoretically interlaced. We thought how these ‘ingredients’ can be described within a dichotomy between the experience of ‘Space’. We used the ‘Space’ imagery to describe a vigilant expectation patients expressed to have their own physical and psychological ‘space’ where they are being ‘seen’ by their providers (Table 1). The ‘Space’ is both *physical* (being able get an appointment; having an unrushed visit to be able to tell their story), but also *psychological* (being acknowledged as a unique, equal, respected, whole person; being authentically, warmly and compassionately cared; being heeded). When these fundamental human needs are unmet, the immediate experience is feeling ‘translucent’, invisible, unheard, unimportant, ignored, patronized and overlooked; feeling treated as a set of clinical symptoms instead of a whole person; as a ‘number’ instead of a named person; feeling rushed; feeling that providers do not really care about them; or that they can’t see the same clinician overtime.

**Table 1:** **‘Ingredients’ of person-centeredness as a dichotomy between experiences of absence and lack of ‘Space’**

	**Absence of ‘Space’**	**Lack of ‘Space’**
“*Holism*”	My providers acknowledge me as a whole person	My providers treat me as a set of clinical symptoms instead of a whole person
“*Naming*”	My providers acknowledge me as a unique, named, equal, respected person, and treat me with respect and dignity; I am seen and heard	I feel a ‘number’ instead of a named person; I feel unimportant, ignored, patronized, overlooked, unseen, unheard
“*Heed*”	*My providers listen and pay heed to me; they allow me enough time to be able to speak, to tell my unique story; they provide me with comprehensive clinical judgment.*	I am rushed to finish the appointment
“*Compassion*”	My providers care for me authentically, warmly and compassionately	My providers don’t really care about me
“*Agency and Empowerment*“	I am involved in care, I am informed, in control, and able to make decisions.	I feel a passive recipient of care
“*Continuity of care*”	I see the same clinician every time each time I come; someone knows about me	I can’t see the same clinician each time; nobody knows about me

## Discussion

Aiming to explore patient narratives related to person-centeredness in the integrated care context, we thematized six ‘ingredients’ of person-centeredness. While we tried here to separate the fine ‘ingredients’ of person-centeredness, patients do not necessarily distinguish between them. The experience of person-centeredness as portrayed here, can be simplified into basic psychological needs of being acknowledged, respected, understood, seen and heard, that can be encompassed as a continuum between the feeling of having emotional and physical ‘Space’ vs. lack of such a space.

### How our findings correspond with the literature on person-centeredness, and how can they be framed within a sociological perspective?

The elements describe here are conceptually similar to other meanings of what person-centeredness constitutes [2-9]. Issues around access, consultation length, provision of information and communication (e.g. feeling that their GP do not always listen to them or consider their opinions seriously) are well known in the literature [39]. Elements of the current healthcare provision such as the rushed, impersonal, segregated nature of the doctor-patient relationship, are frustrating for diabetic patients [50]. In addition, limited consultation time is an important barrier to patient-centered care [51].

What is unique about these narratives is that they are brought here in the context of integrated care. ‘Person-centeredness’ in the context of integrated care may have different meanings to as those in other contexts, specifically the traditional non-integrated context. In the non-integrated context, the six ‘ingredients’ of person-centeredness that emerged from our data can be enacted by individual clinicians, yet without spanning as a systemic ethos across different services and providers serving the patient.

For example, current typologies of the patient-physician encounter usually refer to the patient-physician encounter as a ‘dyad’ [52]. But do the same assumptions on the patient-physician encounter, as well as patient and physician roles, retain when patients meet with multiple, supposedly coordinate clinicians? Does it matter whether these physically-fragmented clinicians liaise with others regarding the specific patient, forming a coordinated, integrated care? Integrated care conceptually extends the ‘dyad’ to a ‘triad’, ‘quad’ and so on, where several providers liaise with a patient. Is it indeed a ‘triad’, for example, or yet two ‘dyads’? Do properties of a ‘dyad’ retain in allegedly integrated ‘triads’? So or so, talking about person-centeredness in the integrated care context call for theoretical treatment of how these two seemingly-related concepts theoretically correspond with each other, and how integrated care affect the traditional typologies of the patient-physician encounter.

Second, patient narratives brought here add some fine nuances, such as the meanings patients attributed to ‘care’. Current approaches to the delivery of care for patients with long-term conditions emphasize more high-level public health policy imperatives, such as promoting prevention, managing the political environment, building integrated health care, effective use of information technologies etc., [10,45], however, they lack a literal mention of compassion as core element of medical care. Above all, authentic compassion – to really *care* about patients as persons, was probably the most missing element in the patient experience of care as portrayed here.

### Towards person-centered integrated care: integrating the patient into integrated care

It seems that patients with positive experiences were fortunate to be treated by person-centered providers rather than person-centeredness implemented systemically. Yet fully developed integrated care in its broadest manifestation requires person-centeredness. Person-centeredness requires an attitudinal shift: patients should be seen as the ‘subject’ of integrated care, the focal point around which services are integrated, instead of a passive ‘object’ that receives a set of integrated services. We cautiously suggest, based on our findings, that before patients want their care to be ‘integrated’ (in the way the system perceives integration), they want it to be person-centered. From the patient perspective, integrated care without person-centeredness is no more integrated than the current models. As others, we suggest that for person-centeredness to become a core element in treating long-term conditions, it should be applied conscientiously and systematically [53], and that the integrated care needs to be introduced in a person-centered way that view patients as a center of integrated care [40].

Patient narratives provide valuable insights into person-centric elements that can be implemented in integrated care programs. Clinicians’ attitude towards the patient as a whole, unique, respected person, and attention to patients psychological needs, in a compassionate and empowering encounter, can breed satisfaction, engagement, trust and adherence with therapy, that altogether can improve clinical outcomes [54]. Making sure patients are able to see the same clinician over time can help both patients and clinicians to create trusted relationship and a ‘therapeutic rapport’ essential for the treatment of long-term conditions. This continuity of care is highly valued by patients [55] and is related with improved outcomes [56-58] and increased patient satisfaction. [59] Longer visits allowing patients to cover range of concerns and the physician to properly attend these concerns, creates that physical and psychological space invaluable to patients. Longer visits should not necessarily mean extended costs for the system or delayed access to other patients. On the contrary, they can potentially save time and costs by allowing clinicians to properly attend to patients’ concerns, and advise on the most appropriate course of treatment, further tests and further psychological support if needed.

### Limitations

A limitation to be acknowledged is that the qualitative data collected through the semi-structured interviews represent narratives of a purposive, non-randomized sample of patients. Hence those agreed to be interviewed may have been driven by either positive or negative experiences with the pilot. The narratives brought here represent views of patients from a specific borough, yet patients from other boroughs may have had different experiences. We employed this recruitment approach because we did not have access to the patients’ demographic data and contact details. Second, the interviews were conducted through the first year of the pilot, and hence integration mechanisms were still in an evolving implementation and shaping process. Being a new concept for both patients and providers, possibly intentions to deliver person-centered care were too early to be provisioned and be noticed by patients.

## Conclusions

We aimed to explore patient narratives of person-centeredness in the integrated care context. We described specific elements that can reflect what person-centeredness means for patients in the integrated care context. We themathized the experience of ‘Space’ as an overarching theme portraying patient experience of having their own physical and psychological space vs. feeling ‘translucent’, ‘unseen’ and unheard. Patients want to be ‘seen’ and heard, to feel valued and acknowledged, yet these needs are inconsistently unmet. Both ‘person-centeredness’ and ‘integrated care’ are new concepts to the way care is provisioned and the way patients and providers communicate with each other. They are more than simple technical, organizational changes but conceptual evolvements. Chaining person-centeredness to the integrated care locomotive (or vice versa) can provide positive opportunities to improve both models together and apart. Attention to patients as a focal point-of-care of integrated care is important to achieve better patient engagement in integrated care. Implementing person-centeredness as a core element an integrated care model apparently requires substantial and deep conceptual change in care model, on top of organizational changes.

## References

[1] Ishikawa H, Hashimoto H, Kiuchi T (2013). The evolving concept of “patient-centeredness” in patient–physician communication research. Soc Sci Med.

[2] Levenstein JH, McCracken EC, McWhinney IR, Stewart MA, Brown JB (1986). The patient-centred clinical method. 1. A model for the doctor-patient interaction in family medicine. Fam Pract.

[3] Brown J, Stewart M, McCracken E, Mcwhinney IR, Levenstein J (1986). The patient-centred clinical method. 2. Definition and application. Fam Pract.

[4] Mead N, Bower P, Hann M (2002). The impact of general practitioners’ patient-centredness on patients’ post-consultation satisfaction and enablement. Soc Sci Med.

[5] Mead N, Bower P (2000). Patient-centredness: a conceptual framework and review of the empirical literature. Soc Sci Med.

[6] Little P, Everitt H, Williamson I, Warner G, Moore M, Gould C, Ferrier K, Payne S (2001). Observational study of effect of patient centredness and positive approach on outcomes of general practice consultations. BMJ.

[7] Bechtel C, Ness DL (2010). If you build it, will they come? Designing truly patient-centered health care. Health Aff.

[8] Leplege A, Gzil F, Cammelli M, Lefeve C, Pachoud B, Ville I (2007). Person-centredness: conceptual and historical perspectives. Disabil Rehabil.

[9] Hudon C, Fortin M, Haggerty JL, Lambert M, Poitras M-E (2011). Measuring patients’ perceptions of patient-centered care: a systematic review of tools for family medicine. Ann Fam Med.

[10] Institute of Medicine. Committee on Quality of Health Care in America (2001). Crossing the Quality Chasm: A New Health System for the 21st Century.

[11] Aarts JWM, Huppelschoten AG, van Empel IWH, Boivin J, Verhaak CM, Kremer JAM, Nelen WL (2012). How patient-centred care relates to patients’ quality of life and distress: a study in 427 women experiencing infertility. Hum Reprod.

[12] Kinmonth AL, Woodcock A, Griffin S, Spiegal N, Campbell MJ (1998). Randomised controlled trial of patient centred care of diabetes in general practice: impact on current wellbeing and future disease risk. BMJ.

[13] Greenfield S, Kaplan SH, Ware JE, Yano EM, Frank HJ (1988). Patients’ participation in medical care: effects on blood sugar control and quality of life in diabetes. J Gen Intern Med.

[14] Vermeire E, Hearnshaw H, Rätsep A, Levasseur G, Petek D, van Dam H, van der Horst F, Vinter-Repalust N, Wens J, Dale J, Van Royen P (2007). Obstacles to adherence in living with type-2 diabetes: an international qualitative study using meta-ethnography (EUROBSTACLE). Prim Care Diabetes.

[15] Van Dam HA, van der Horst F, van den Borne B, Ryckman R, Crebolder H (2003). Provider-patient interaction in diabetes care: effects on patient self-care and outcomes. A systematic review. Patient Educ Couns.

[16] Kaplan SH, Greenfield S, Ware JE (1989). Assessing the effects of physician-patient interactions on the outcomes of chronic disease. Med Care.

[17] Stewart MA (1995). Effective physician-patient communication and health outcomes: a review. CMAJ.

[18] Charles C, Gafni A, Whelan T (1997). Shared decision-making in the medical encounter: what does it mean? (or it takes at least two to tango). Soc Sci Med.

[19] Thompson AGH (2007). The meaning of patient involvement and participation in health care consultations: a taxonomy. Soc Sci Med.

[20] Boyer CA, Lutfey KE (2010). Examining critical health policy issues within and beyond the clinical encounter: patient-provider relationships and help-seeking behaviors. J Health Soc Behav.

[21] Potter SJ, McKinlay JB (2005). From a relationship to encounter: an examination of longitudinal and lateral dimensions in the doctor-patient relationship. Soc Sci Med.

[22] Timmermans S, Oh H (2010). The continued social transformation of the medical profession. J Health Soc Behav.

[23] Fox NJ, Ward KJ, O’Rourke AJ (2005). The “expert patient”: empowerment or medical dominance? The case of weight loss, pharmaceutical drugs and the Internet. Soc Sci Med.

[24] Anderson RM, Funnell MM (2005). Patient empowerment: reflections on the challenge of fostering the adoption of a new paradigm. Patient Educ Couns.

[25] Emanuel EJ, Emanuel LL (1992). Four models of the physician-patient relationship. JAMA.

[26] Teutsch C (2003). Patient-doctor communication. Med Clin North Am.

[27] Holmström I, Röing M (2010). The relation between patient-centeredness and patient empowerment: a discussion on concepts. Patient Educ Couns.

[28] Ham C, Curry N (2011). Integrated Care Summary: What Is It? Does It Work? What Does It Mean for the NHS?.

[29] Kodner DL, Spreeuwenberg C (2002). Integrated care: meaning, logic, applications, and implications–a discussion paper. Int J Integr Care.

[30] Leichsenring K (2004). Developing integrated health and social care services for older persons in Europe. Int J Integr Care.

[31] Mezzich JE, Snaedal J, van Weel C, Heath I: **Introduction to conceptual explorations on person-centered medicine.** *Int J Integr Care* 2010, **10**. 10.5334/ijic.472 PMC2834893 20228867

[32] Olsson L-E, Hansson E, Ekman I, Karlsson J (2009). A cost-effectiveness study of a patient-centred integrated care pathway. J Adv Nurs.

[33] Briggs CJ, Capdegelle P, Garner P: **Strategies for integrating primary health services in middle- and low-income countries: effects on performance, costs and patient outcomes.** *Cochrane Database Syst Rev* 2001, (4):CD003318. 10.1002/14651858.CD003318 11687187

[34] Ouwens M, Wollersheim H, Hermens R, Hulscher M, Grol R (2005). Integrated care programmes for chronically ill patients: a review of systematic reviews. Int J Qual Health Care.

[35] Amati F, McDonald AM, Majeed A, Dubois E, Rawaf S (2012). Implementation and evaluation of patient centred care in experimental studies from 2000-2010: systematic review. International Journal of Person Centered Medicine.

[36] Nolte E, McKee M (2008). Caring For People With Chronic Conditions: A Health System Perspective: A Health System Perspective.

[37] **Equity and excellence: liberating the NHS (White Paper).** [http://www.dh.gov.uk/en/Publicationsandstatistics/Publications/PublicationsPolicyAndGuidance/DH_117353]

[38] **Liberating the NHS: No decision about me, without me.** [https://www.gov.uk/government/uploads/system/uploads/attachment_data/file/216980/Liberating-the-NHS-No-decision-about-me-without-me-Government-response.pdf]

[39] Coulter A, Elwyn G (2002). What do patients want from high-quality general practice and how do we involve them in improvement?. Br J Gen Pract.

[40] Roland M, Lewis R, Steventon A, Abel G, Adams J, Bardsley M, Brereton L, Chitnis X, Conklin A, Staetsky L, Tunkel S, Ling T (2012). Case management for at-risk elderly patients in the English Integrated Care Pilots: observational study of staff and patient experience and secondary care utilisation. Int J Integr Care.

[41] Gulmans J, Vollenbroek-Hutten MMR, Van Gemert-Pijnen JEWC, Van Harten WH (2009). Evaluating patient care communication in integrated care settings: application of a mixed method approach in cerebral palsy programs. Int J Qual Health Care.

[42] Walker KO, Labat A, Choi J, Schmittdiel J, Stewart AL, Grumbach K (2013). Patient perceptions of integrated care: confused by the term, clear on the concept. Int J Integr Care.

[43] Bastiaens H, Van Royen P, Pavlic DR, Raposo V, Baker R (2007). Older people’s preferences for involvement in their own care: a qualitative study in primary health care in 11 European countries. Patient Educ Couns.

[44] Yach D, Hawkes C, Gould CL, Hofman KJ (2004). The global burden of chronic diseases: overcoming impediments to prevention and control. JAMA.

[45] **Innovative Care for Chronic Conditions: Building Blocks for Action.** [http://www.who.int/chp/knowledge/publications/icccreport/en/]

[46] Curry N, Harris M, Gunn L, Pappas Y, Blunt I, Soljak M, Mastellos N, Holder H, Smith J, Majeed A, Ignatowicz A, Greaves F, Belsi A, Costin-Davis N, Nielsen JDJ, Greenfield G, Cecil E, Patterson S, Car J, Bardsley M (2013). Integrated care pilot in North West London: a mixed methods evaluation. Int J Integr Care.

[47] Harris M, Greaves F, Patterson S, Jones J, Pappas Y, Majeed A, Car J (2012). The North West London Integrated Care Pilot: innovative strategies to improve care coordination for older adults and people with diabetes. J Ambul Care Manage.

[48] Green J, Thorogood N (2009). Qualitative Methods for Health Research.

[49] Glaser BG, Strauss AL (2008). The Discovery of Grounded Theory: Strategies for Qualitative Research.

[50] Ciechanowski P, Katon WJ (2006). The interpersonal experience of health care through the eyes of patients with diabetes. Soc Sci Med.

[51] Dunn N (2003). Practical issues around putting the patient at the centre of care. JRSM.

[52] Kenny DA, Veldhuijzen W, Van der Weijden T, LeBlanc A, Lockyer J, Légaré F, Campbell C (2010). Interpersonal perception in the context of doctor–patient relationships: A dyadic analysis of doctor–patient communication. Soc Sci Med.

[53] Ekman I, Swedberg K, Taft C, Lindseth A, Norberg A, Brink E, Carlsson J, Dahlin-Ivanoff S, Johansson I-L, Kjellgren K, Lidén E, Öhlén J, Olsson L-E, Rosén H, Rydmark M, Sunnerhagen KS (2011). Person-centered care–ready for prime time. Eur J Cardiovasc Nurs.

[54] Stewart M, Brown JB, Donner A, McWhinney IR, Oates J, Weston WW, Jordan J (2000). The impact of patient-centered care on outcomes. J Fam Pract.

[55] Aboulghate A, Abel G, Elliott MN, Parker RA, Campbell J, Lyratzopoulos G, Roland M (2012). Do English patients want continuity of care, and do they receive it?. Br J Gen Pract.

[56] Huntley A, Lasserson D, Wye L, Morris R, Checkland K, England H, Salisbury C, Purdy S (2014). Which features of primary care affect unscheduled secondary care use? A systematic review. BMJ Open.

[57] Saultz JW, Lochner J (2005). Interpersonal continuity of care and care outcomes: a critical review. Ann Fam Med.

[58] Hsiao C-J, Boult C (2008). Effects of quality on outcomes in primary care: a review of the literature. Am J Med Qual.

[59] Wasson JH, Sauvigne AE, Mogielnicki RP, Frey WG, Sox CH, Gaudette C, Rockwell A (1984). Continuity of outpatient medical care in elderly men. A randomized trial. JAMA.

